# The comparison of biomechanical effects of the conventional and bone-borne palatal expanders on late adolescence with unilateral cleft palate: a 3-dimensional finite element analysis

**DOI:** 10.1186/s12903-022-02640-1

**Published:** 2022-12-13

**Authors:** Wen-yu Meng, Yan-qing Ma, Bing Shi, Ren-kai Liu, Xiao-ming Wang

**Affiliations:** 1 Department of Ultrasound Imaging, The First Hospital of Lanzhou University, 730000 Lanzhou, The People’s Republic of China; 2 Key Laboratory of Dental Maxillofacial Reconstruction and Biological Intelligence Manufacturing (NO: 20JR10RA653 - ZDKF20210401), School of Stomatology, Lanzhou University, No. 199, Donggang West Road, Gansu Province 730000 Lanzhou, People’s Republic of China; 3 Department of Orthodontics, School of Stomatology, Lanzhou University, Lanzhou, 730000 Gansu Province People’s Republic of China; 4 State Key Laboratory of Oral Diseases & National Clinical Research Center for Oral Diseases, West China Hospital of Stomatology, Sichuan University, Chengdu, 610041 The People’s Republic of China; 5 Department of Orthodontics and Pediatric Dentistry, West China Stomatological Hospital, West China College of Stomatology, Sichuan University, No. 14, Section 3, Ren Min Nan Road, Chengdu, 610041 People’s Republic of China

**Keywords:** Finite element analysis, Rapid palatal expansion, Miniscrew, Unilateral cleft lip and palate

## Abstract

**Background:**

Patients with unilateral cleft lip and palate were associated with different nasomaxillary complex from the normal population. Although the biomechanical effects of conventional rapid palatal expansion (Hyrax expansion) and bone-borne rapid palatal expansion (micro-implant-assisted expansion) in non-cleft patients have been identified by multiple studies, little is known in patients with unilateral cleft lip and palate. The purpose of this study was to investigate and compare the biomechanical effects of the conventional and bone-borne palatal expanders in a late adolescence with unilateral cleft lip and palate.

**Methods:**

A cone beam CT scan of a late adolescence with unilateral cleft lip and palate was selected to construct the three-dimensional finite element models of teeth and craniofacial structures. The models of conventional and born-borne palatal expanders were established to simulate the clinical maxillary expansion. The geometric nonlinear theory was applied to evaluate the Von Mises stress distribution and displacements in craniofacial structures and teeth.

**Results:**

Bone-borne palatal expander achieved more transverse movement than conventional palatal expander in the whole mount of craniofacial regions, and the maximum amount of expansion was occurred anteriorly along the alveolar ridge on cleft-side. The expanding force from born-borne palatal expander resulted in more advancement in nasomaxillary complex than it in conventional palatal expander, especially in the anterior area of the minor segment of maxilla. Stresses from the both expanders distributed in similar patterns, but larger magnitudes and ranges were generated using the bone-borne expander around the maxillary buttresses and pterygoid plates of sphenoid bone. The maximum expanding stresses from born-borne palatal expander were concentrated on palatal slope supporting minscrews, whereas those from conventional palatal expander were concentrated on the anchoring molars. In addition, the buccal tipping effect of teeth generated using the bone-borne expander was less than it using the conventional palatal expander.

**Conclusion:**

Bone-borne expander generated enhanced skeletal expansion at the levels of alveolar and palate in transversal direction, where the miniscrews contributed increased expanding forces to maxillary buttresses and decreased forces to buccal alveolar. Bone-borne expanders presented a superiority in correcting the asymmetric maxilla without surgical assistant in late adolescence with unilateral cleft lip and palate.

## Introduction

Unilateral cleft lip and palate (UCLP) is the most common craniofacial deformity characterized by the dismissed midpalatal suture and defected alveolar bone, which separates the maxillary structures into the minor and major segments. Postnatal surgical repair is a necessary treatment but causes tremendous soft-tissue tensions from scar contracture. This results with the collapse of the maxillary minor segment with mediolingual rotation, anterior crossbite with or without posterior crossbite, and maxillary growth retardation in transverse and sagittal dimensions at later stages [1, 2]. Thus, rapid palatal expansion and maxillary protraction are routinely adopted in orthodontic treatment to correct the maxillofacial deformity in adolescent period.

For patients with maxillary dysplasia in late adolescence, treatment using the combination of protraction facemask and conventional hyrax expander (tooth-anchored rapid palatal expander, C-RPE) generates limited effect on the correction of sagittal and transverse deficiencies in maxilla [3, 4]. The LeFort I osteotomy on the minor segment and vertical osteotomy on alveolar bone were necessary for eliminating the resistance to expansion [2, 5]. However, numerous studies reported that C-RPE generated approximately 50% tooth effect, which resulted in excessive buccal tipping of the tooth axes, obvious root resorption and decreased buccal bone thickness [6, 7]. Thus, the C-RPE was commonly recommended to use at the pubertal or pre pubertal stage.

In recent years, micro-implant-assisted rapid palatal expansion (bone-borne expander, B-RPE) was gradually applied, which was proved to be effective in expanding the heavily interdigitated sutures without surgical assistance, and generating less dentoalveolar side effects for late adolescence or even adults [6, 8, 9]. By morphological measurements, various studies compared the clinical and mechanical discrepancies between the C-RPE and B-RPE [9–11]. For UCLP patients, because of the separated midpalate and asymmetrical segments of maxilla, the expansive forces from expander and the resistance from circum-maxillary structures were definitely different from the non-cleft [12]. Moreover, unlike the commonly adopted implantation sites in normal patients, the absence of midpalatal suture in UCLP patients restricted the routine implantation places [2, 13]. For the UCLP patients, micro-implant-assisted rapid palatal expansion might allow an enhanced simultaneous expansion of the surrounding hypertrophic scar, which contributed to long-term stability of the expansion, and to reduce the risk of relapse [14]. However, for the late adolescence with UCLP, few literatures reported the application of bone-borne expander. The stress distribution and displacement pattern in mid-palatal area and around craniofacial structures were also not well known.

The finite element analysis (FEA) is a well-proven mathematical technique for calculating the displacement, internal stress and strain in the craniofacial structures during orthodontics [15]. The transmission and dissipation of expansive forces, as well as the deformating features of the nasomaxillary complex can be objectively characterized using FEA [10, 11, 16, 17]. Thus, using finite element analysis in UCLP patient, the purpose of this study is to investigate the discrepancy of biomechanical effects on the craniofacial structures between C-RPE and B-RPE. Our research will provide guidance for the suitable design and application of B-RPE in late adolescence with UCLP.

## Materials and methods

### CBCT imaging and finite element modeling

The CBCT data saved as DICOM-format was selected from an 18-year-old adolescence with UCLP, presenting narrow arch and complete dental crossbite. The CBCT was taken for the examination of pre-orthodontic (Smart3D-X, parameters for image acquisition were 100 kV, 6 mA, 26.9 s, field of view of 23 × 18 cm, and voxel size of 0.25 mm), approved by the Ethic Committee of School of Stomatology, Lanzhou University (Approval No. LZUKQ-2022-028). All experiments were performed in accordance with relevant guidelines and regulations.

**Fig. 1:**
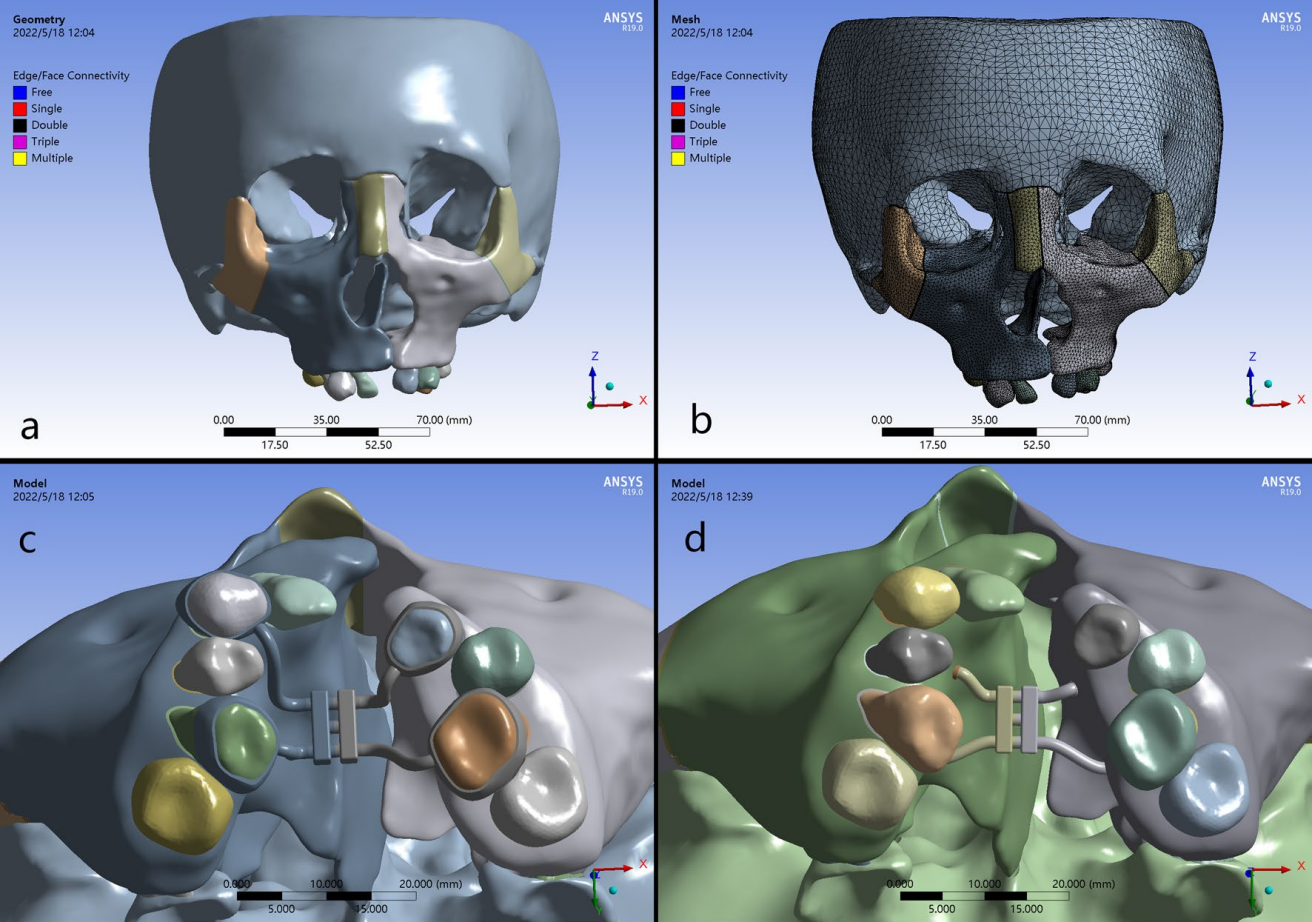
Geometrical models of the craniofacial structures and rapid maxillary expanders of the patient with UCLP: **a** Construction of skull model with sutures; **b** finite element meshes of the craniofacial structures in different sizes of tetrahedrons; **c** C-RPE, the conventional tooth-borne rapid maxillary expander (Hyrax expander); **d** B-RPE, bone-borne rapid palatal expander anchored by micro-implants (8.5 mm length, 1.8 mm diameter) on the palatal slope, with the miniscrews 8 mm beneath the alveolar ridge, between the first and second premolars (the anterior part), the first and second molars (the posterior part)

The CBCT data was imported into MIMICS software (version 19.0; Materialise, Leuven, Belgium), in which the craniofacial structures and teeth were seperated. Subsequently, files in STL format were transformed and processed in Geomagic Studio (Version 2017; Raindrop, USA) to generate the high-quality surface meshes. The sutures and PDL were sectioned into 0.2-mm tetrahedrons; the maxilla, dentition, and alveolar bone in the volumetric meshes were sectioned into 1.5-mm tetrahedrons; the remaining skull bones including surrounding sutures were sectioned into 4-mm tetrahedrons using ANSYS 19.0 (ANSYS Inc., Canonsburg, PA, USA). Finally, three dimensional meshes were composed of 1,186,791 tetrahedral elements and 1,806,003 nodes (Fig. 1a–b).

### Design of the expanders

The design of the conventional hyrax expander model was made as reported by Lee et al. [10]. The bone-borne expander was connected by an expansion screw (0.25 mm widening per turn) and stainless steel wires (diameter 1.0 mm), and supported by 4 miniscrews (C-implants; Cimplant, Seoul, Korea; length 8.5 mm; diameter 1.8 mm). Both of the appliances were placed parallelly to the mid-palate in anterior-posterior direction, as close to the palate as possible. All the miniscrews were placed at the palatal slopes, 8 mm beneath the alveolar ridge, from the first and second premolars (the anterior part) to the first and second molars (the posterior part) [18] (Fig. 1c–d).

### Finite element simulation

**Fig. 2:**
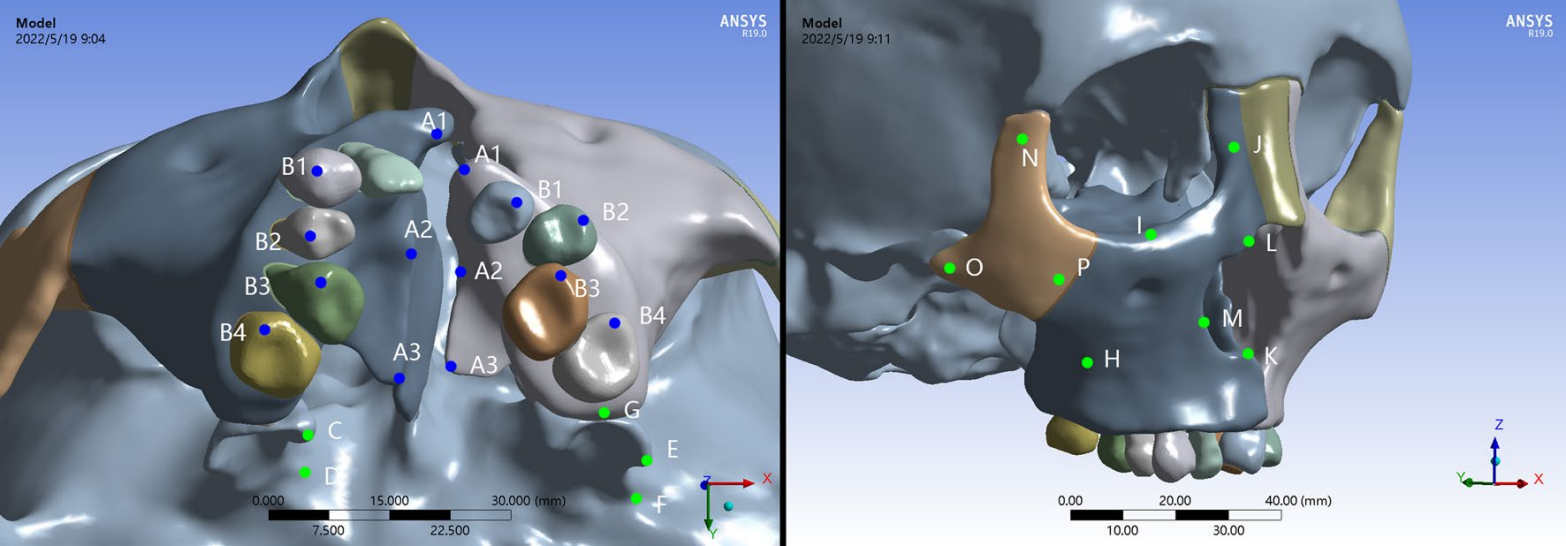
Evaluating landmarks: A1, points of the most anterior region of palate and alveolar in cleft and non-cleft sides; A3, points of the most posterior region of palate in cleft and non-cleft sides; A2, the middle points of A1-A3 line on the palatal ridge in cleft and non-cleft sides; B1–B4, the buccal tips of the first and second pre-molars, and the mesiobuccal tips of the first and second molars in cleft and non-cleft sides; C, superior rim of medial pterygoid plate; D, inferior rim of medial pterygoid plate; E, superior rim of lateral pterygoid plate; F, inferior rim of lateral pterygoid plate; G, maxillary tuberosity; H, maxillary anterior border; I, inferior orbital rim of maxilla J, frontal process of maxilla; K, anterior nasal spine; L, superior rim of nasal cavity M, inferior rim of nasal cavity; N, frontal process of zygomatic bone; O, temporal process of zygomatic bone; P, maxillary process of zygomatic bone

In ANSYS 19.0 software, the mechanical properties of the materials in the model were assigned according to the previous studies, as shown in Table 1 [10, 12, 19]. The teeth, craniofacial bone, sutures, periodontal ligament, mini-screws and expanders were considered to be homogenous and isotropic. The thickness of the maxillofacial sutures and periodontal ligament were 0.5 and 0.2 mm as previous studies shown, respectively [8, 10, 17]. The foramen magnum was completely fixed without any freedom and used as the origin point. The 3D coordinates were X, transverse plane; Y, sagittal plane; and Z, vertical plane, as suggested by previous studies[10, 17, 20]. Positive values indicate the cleft side, backward, and upward displacements on the X, Y, and Z planes. Expanders were activated transversely for 0.25 mm displacement at the level of the expansion screws to simulate the clinical daily expansion [12, 20]. The expanders were banded to the teeth or minicrews in both groups. For FEA, displacements, von Mises stresses, and shear stress in marked regions were performed and evaluated (Fig. 2).

**Table 1:** Material properties

Material	Young’s modulus (MPa)	Poisson’s ratio
Alveolar bone and palate	13,700	0.30
Tooth	19,890	0.31
Periodontal ligament	50	0.49
Suture	10	0.49
Expander	190,000	0.33
Miniscrew	113,000	0.33

## Results

### Displacement of the palatal bone

**Fig. 3:**
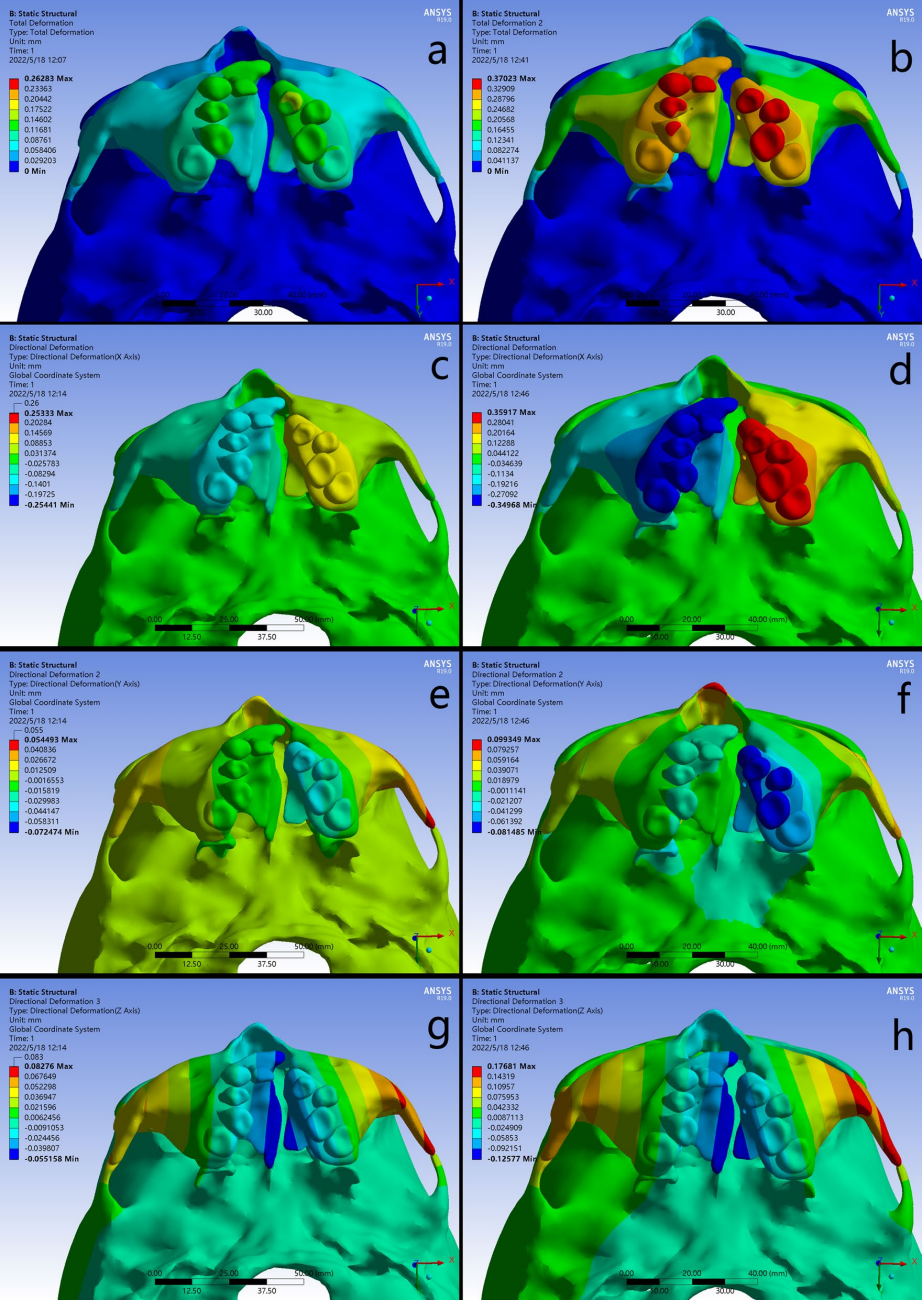
Displacement patterns of palate and teeth in occlusal views. **a**-**b** the total displacement, **c**-**d** the transversal movement on X axis, **e**-**f** the sagittal movement on Y axis, **g**-**h** the vertical movement on Z axis

The three dimensional displacements of the palatal reference points along the anterior, middle and posterior edges were respectively shown in Table 2. The maximum total displacement of palate was observed in the anterior area, and presented decreasing pattern toward the posterior on cleft and non-cleft sides. The B-RPE generated larger amount of deformation than the C-RPE in corresponding palatal regions. In addition, the displacements were generally greater on cleft side than non-cleft side (Fig. 3a–b).

**Table 2:** Displacements (mm) in palate and teeth after activation of the C-RPE and B-RPE appliances (mm)

Region	C-RPE								B-RPE							
	Cleft side				Non-cleft side				Cleft side				Non-cleft side			
	Total	X	Y	Z	Total	X	Y	Z	Total	X	Y	Z	Total	X	Y	Z
Anterior palate (A1)	0.1326	0.1236	− 0.0348	− 0.0320	0.1230	− 0.1126	− 0.0150	− 0.0459	0.3174	0.3018	− 0.0674	− 0.0751	0.3036	− 0.2817	− 0.0088	− 0.1032
Middle palate (A2)	0.1000	0.0889	− 0.0275	− 0.0377	0.0926	− 0.0810	− 0.0101	− 0.0404	0.2574	0.2351	− 0.0516	− 0.0868	0.2479	− 0.2309	− 0.0009	− 0.0922
Posterior palate (A3)	0.0912	0.0698	− 0.0277	− 0.0515	0.0742	− 0.0595	− 0.0078	− 0.0401	0.2441	0.2085	− 0.0503	− 0.1206	0.2134	− 0.1960	0.0038	− 0.0906
Cusp tip of 4 (B1)	0.1380	0.1435	− 0.0363	− 0.0157	0.1485	− 0.1368	− 0.0107	− 0.0060	0.3693	0.3583	− 0.0772	− 0.0358	0.3498	− 0.3492	− 0.0066	− 0.0138
Cusp tip of 5 (B2)	0.1299	0.1383	− 0.0255	0.0044	0.1405	− 0.1294	− 0.0076	− 0.0064	0.3508	0.3457	− 0.0630	0.0091	0.3404	− 0.3400	− 0.0054	− 0.0091
Cusp tip of 6 (B3)	0.1260	0.1362	− 0.0289	− 0.0093	0.1403	− 0.1252	− 0.0074	− 0.0103	0.3539	0.3460	− 0.0695	− 0.0232	0.3339	− 0.3324	− 0.0054	− 0.0196
Cusp tip of 7 (B4)	0.1137	0.1145	− 0.0175	0.0063	0.1164	− 0.1137	− 0.0028	0.0028	0.3118	0.3065	− 0.0488	0.0171	0.3118	− 0.3113	0.0009	0.0157

Figure 2 presented the definitions of each landmark. Transverse displacement: (+) cleft expansion (–) non-cleft expansion; Sagittal displacement, (-) forward, (+) backward; Vertical displacement: (+) upward, (–) downward

**Fig. 4:**
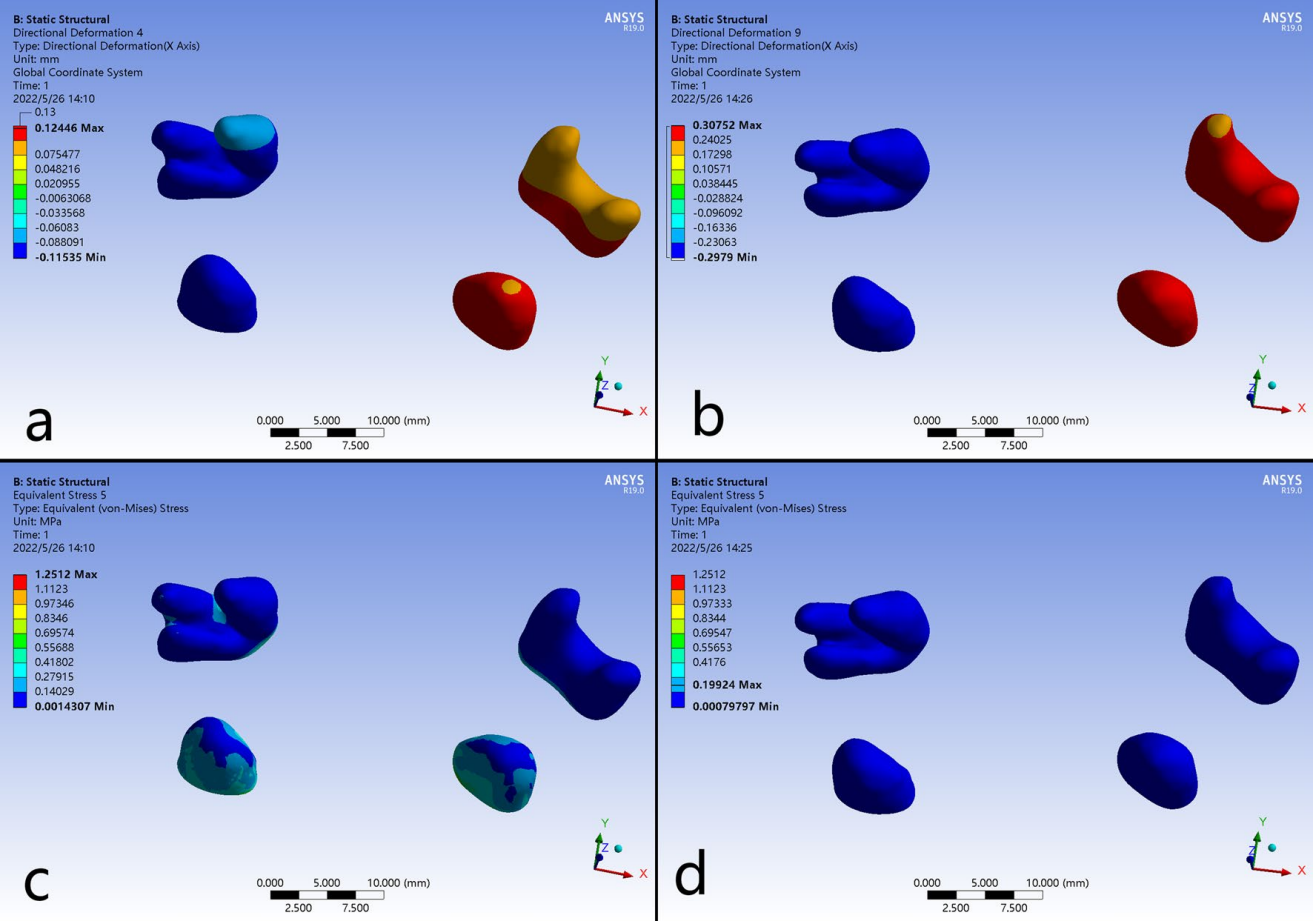
Displacement patterns and stress distributions of periodontal ligaments around the bilateral first pre-molars and first molars. **a**, **c** C-RPE, **b**, **d** B-RPE

Transversally on the X axis, compared with C-RPE, B-RPE presented an obviously greater displacement on both the cleft and non-cleft sides. The transversal dispalcements were gradually decreased from the anterior to the posterior, presenting a pyramidal expanding (Fig. 3c–d). Sagittally on the Y axis, the forward displacements of palate on cleft and non-cleft sides were obviously greater in B-RPE than those in C-RPE. The both appliances generated greater forward displacements on cleft side than non-cleft side, and the maximum value concentrated on the anterior region on cleft sides (Fig. 3e–f). Vertically on the Z axis, the bilateral palatal bone presented inferior displacement in both groups along the anterior-posterior axis, of which the amount was significantly increased in B-RPE compared with the C-RPE (Fig. 4g–h).

### Displacement of the teeth

The three dimensional displacements of the cusp tips of pre-molars and molars were shown in Table 2. In contrast to C-RPE, the amount of the total displacement on cleft side was generally greater than it on non-cleft side, indicating an asymmetric movement of dental arch in the B-RPE. Interestingly, compared with the C-RPE, B-RPE occurred more remarkable displacement in the corresponding teeth, and the maximum displacement concentrated on the first pre-molar on cleft side (Fig. 3a–b).

Transversally on the X axis, the B-RPE presented greater expansion than C-RPE, and the maximum displacement was occurred at the tip of first pre-molar on cleft side. Moreover, at periodontal ligament level of the first premolar and molar, the displacement in B-RPE occurred more uniformly than it in C-RPE, indicating less tooth effect generated by the bone-borne expander (Figs. 3e-f and 4). Sagittally on the Y axis, both expanders represented forward movement in all the teeth, but with a gradual attenuation from the anterior to posterior (Fig. 3e–f). Vertically on the Z axis, the teeth occurred extrusion or intrusion irregularly (Fig. 3g–h).

### Displacement of the craniofacial bone

**Fig. 5:**
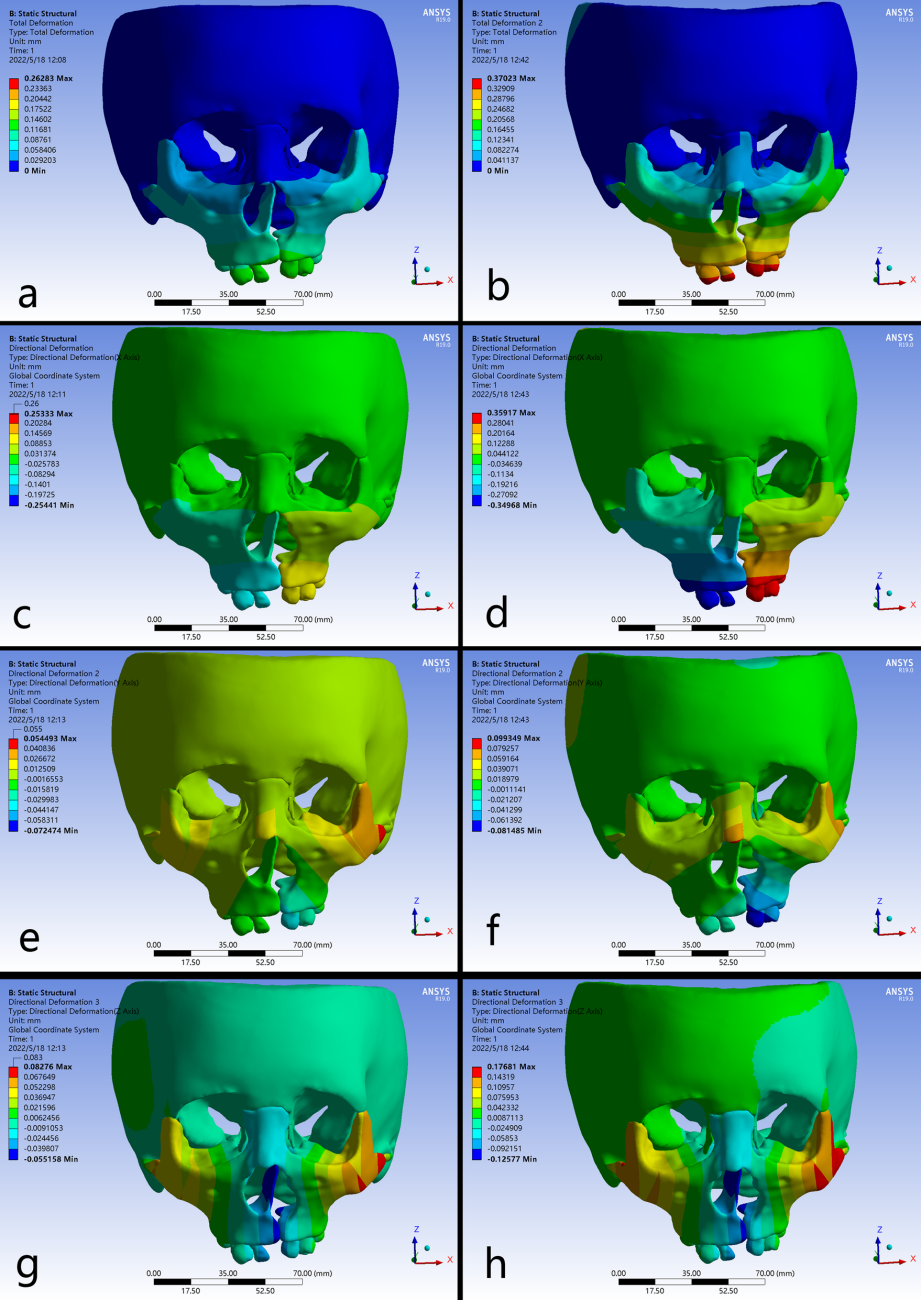
Displacement patterns of craniofacial structures in frontal views. **a**-**b** the total displacement, **c**-**d** the transversal movement on X axis, **e**-**f** the sagittal movement on Y axis, **g**-**h** the vertical movement on Z axis

The displacement in craniofacial structures was shown in Table 3. Both appliances presented similar displacement tendency, but the magnitudes were generally greater in B-RPE than C-RPE. The maximum total displacement presented in a feature that decreased gradually from the dentoalveolar level upward to the skull level, passing through the zygomatic process of frontal bone, zygomatic process of temporal bone, and frontal process of the maxilla. In the both appliances, regions in cleft side generally occurred greater displacement than the corresponding regions in non-cleft side. Moreover, the bone-borne expander generated far-reaching effects on the lateral cranial vault. No significant displacements were detected around the frontal bone and frontonasal suture in both expanders (Fig. 5a–b).

**Table 3:** Maxillofacial landmarks displacements after activation of the C-RPE and B-RPE appliances (mm)

Region	C-RPE								B-RPE							
	Cleft side				Non-cleft side				Cleft side				Non-cleft side			
	Total	X	Y	Z	Total	X	Y	Z	Total	X	Y	Z	Total	X	Y	Z
*Medial pterygoid*																
Superior (C)	0.0106	0.0088	− 0.0049	− 0.0017	0.0238	− 0.0206	− 0.0091	− 0.0038	0.0534	0.0303	− 0.0172	− 0.0059	0.0667	− 0.0642	− 0.0259	− 0.0119
Inferior(D)	0.0009	0.0009	− 0.0005	− 0.0002	0.0019	− 0.0021	− 0.0006	0.0002	0.0039	0.0039	− 0.0018	− 0.0009	0.0080	− 0.0085	− 0.0021	− 0.0009
*Lateral pterygoid*																
Superior (E)	0.0079	0.0065	0.0007	0.0040	0.0133	− 0.0105	− 0.0005	0.0075	0.0266	0.0213	0.0016	0.0133	0.0551	− 0.0493	0.0024	0.0268
Inferior(F)	0.0013	0.0007	0.0003	0.0013	0.0036	− 0.0016	0.0012	0.0031	0.0050	0.0026	0.0011	0.0041	0.0105	− 0.0755	0.0040	0.0089
*Maxilla*																
Maxillary tuberosity(G)	0.0819	0.0820	− 0.0120	− 0.0012	0.0841	− 0.0872	− 0.0005	0.0011	0.2425	0.2359	− 0.0328	− 0.0158	0.2492	− 0.2511	0.0060	0.0078
Anterolateral wall(H)	0.0861	0.0824	− 0.0012	0.0215	0.0879	− 0.0778	0.0062	0.0248	0.2269	0.2073	− 0.0135	0.0652	0.2268	− 0.2181	0.0149	0.0707
Inferior orbital rim(I)	0.0675	0.0334	0.0227	0.0487	0.0510	− 0.0322	0.0129	0.0375	0.1519	0.0932	0.0369	0.1156	0.1334	− 0.0981	0.0302	0.0814
Frontal process(J)	0.0178	0.0015	0.0090	− 0.0141	0.0170	0.0002	0.0048	− 0.0153	0.0482	0.0045	0.0337	− 0.0349	0.0479	− 0.0091	0.0257	− 0.0348
Anterior nasal spine(K)	0.1152	0.1107	− 0.0295	− 0.0285	0.0937	− 0.0839	− 0.0091	− 0.0338	0.2724	0.2648	− 0.0559	− 0.0696	0.2296	− 0.2180	0.0018	− 0.0732
*Nasal cavity*																
Superior rim (L)	0.0427	0.0402	0.0001	− 0.0091	0.0378	− 0.0354	0.0032	− 0.0115	0.1036	0.0916	0.0116	− 0.0203	0.1012	− 0.0993	0.0227	− 0.0269
Inferior rim(M)	0.0888	0.0855	− 0.0153	− 0.0105	0.0793	− 0.0807	− 0.0034	− 0.0153	0.2312	0.2347	− 0.0383	− 0.0233	0.2040	− 0.2080	0.0087	− 0.0321
*Zygomatic bone*																
Frontal process (N)	0.0660	− 0.0077	0.0307	0.0566	0.0465	− 0.0103	0.0118	0.0445	0.1466	− 0.0064	0.0616	0.1372	0.1255	− 0.0411	0.0204	0.1156
Temporal process(O)	0.0869	0.0187	0.0427	0.0729	0.0647	− 0.0308	0.0197	0.0533	0.1988	0.0827	0.0666	0.1668	0.1789	− 0.1120	0.0316	0.1340
Maxillary process(P)	0.0787	0.0469	0.0314	0.0539	0.0611	− 0.0442	0.0140	0.0379	0.1919	0.1306	0.0463	0.1552	0.1695	− 0.1398	0.0229	0.0986

Figure 2 presented the definitions of each landmark. Transverse displacement: (+) cleft expansion (–) non-cleft expansion; Sagittal displacement, (-) forward, (+) backward; Vertical displacement: (+) upward, (–) downward

Transversally on the X axis, the nasomaxillary complex presented a pyramidal expansion with the base of pyramid at the floor of the alveolar ridges and the apex nearby the frontonasal suture. This results of B-RPE were especially obvious. The maximum lateral movement was occurred at the anterior alveolar ridge on cleft side in B-RPE, and greater than that it in C-RPE (Fig. 5c–d). For the cleft and non-cleft segments of maxilla, the expanding was enhanced in the anterior region from the occlusal view in B-RPE. Moreover, the B-RPE caused enhanced lateral bending of the medial and lateral pterygoid plates articulated by the maxillary pterygoid sutures (Fig. 3c–d). Sagittally on the Y axis, the forward displacement of nasomaxillary complex was more remarkable around the medial region in B-RPE than it in C-RPE. By contrast, in zygomatic arch area, B-RPE generated a larger amount of backward displacement on cleft side than C-RPE (Figs. 3e–f and 5e-f). Vertically on the Z axis, the maximum downward displacement was occurred along the marginal areas of cleft palate in both expanders. By contrast, the lateral zygomatic arch presented a remarkable upward displacement, and the maximum value was detected on cleft side in B-RPE (Fig. 3g-h and Fig. 5g–h).

### Stress distribution

**Fig. 6:**
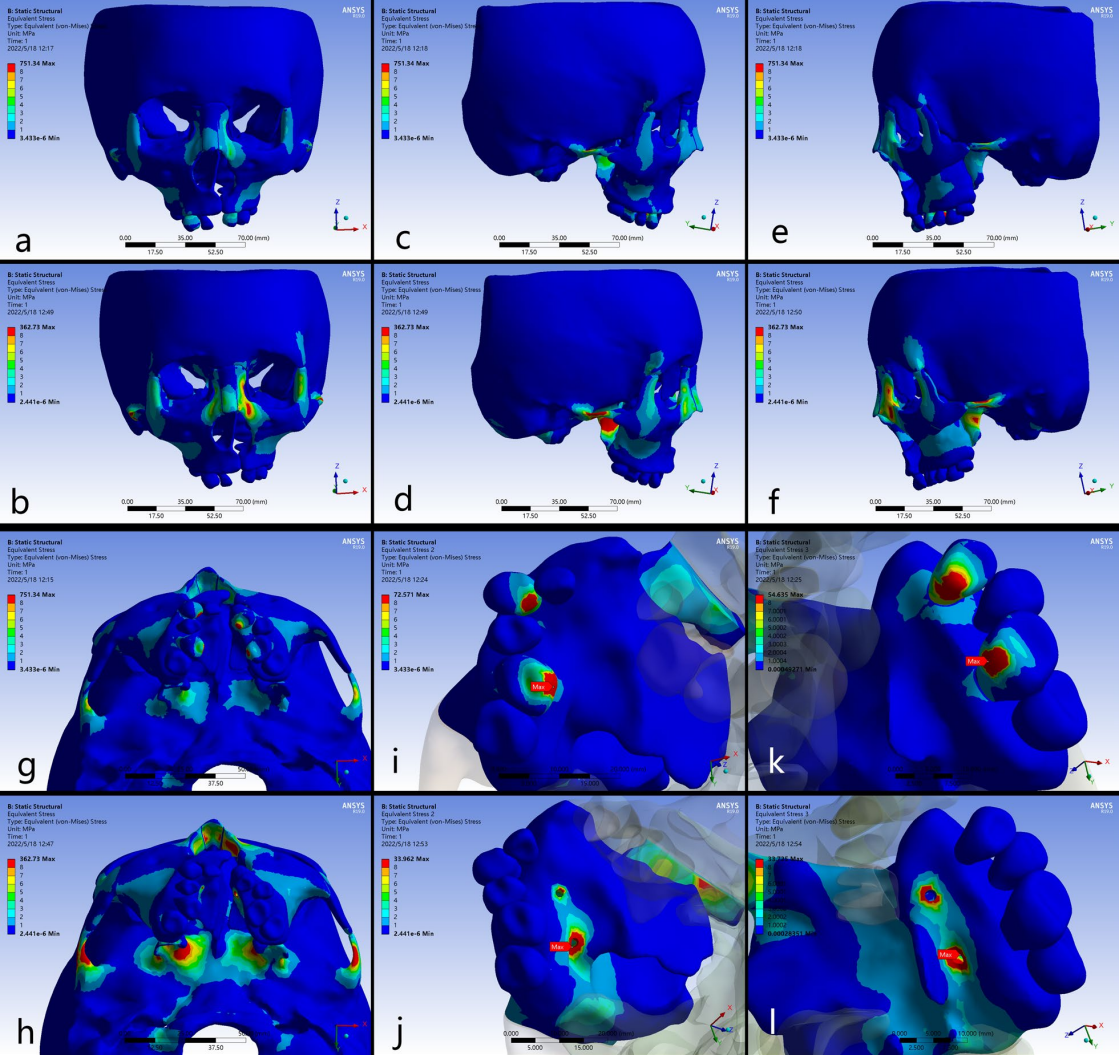
Stress distributions of craniofacial structures. **a**, **c**, **e** frontal and lateral views in C-RPE, **b**, **d**, **f** frontal and lateral views in B-RPE, **g** occlusal view in C-RPE, **h** occlusal view in B-RPE, **i** the non-cleft side of maxilla in C-RPE, **j** the non-cleft side of maxilla in B-RPE, **k** the cleft side of maxilla in C-RPE, **l** the cleft side of maxilla in B-RPE

**Fig. 7:**
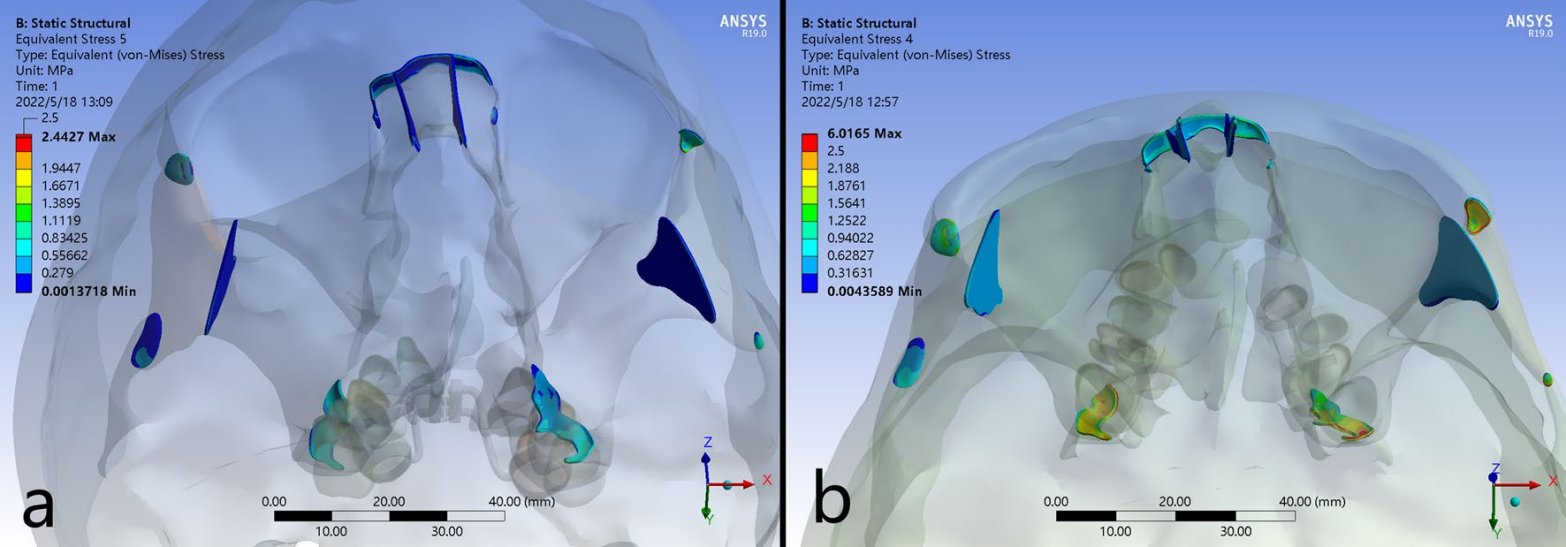
Stress distributions of craniofacial sutures. **a** C-RPE, **b** B-RPE.

The higher stresses exerted from the expanders were distributed in similar patterns around the buttress and lateral borders of maxilla, the inferior and superior borders of nasal cavity, the lateral border of orbits, and the transition regions of pterygoid plates (Tables 4 and 5; Fig. 6). For the maxillofacial bones, higher stresses could be detected in B-RPE compared to those in C-RPE in the corresponding regions (Fig. 6a–h). The maximum stress was concentrated on the lingual crown of anchorage teeth with a value of 72.571 MPa in C-RPE, while it occurred at the implant insertion site with a value of 33.962 MPa in B-RPE (Fig. 6i–l). In addition, the expanding force exerted directly from the palatal slope to the surrounding craniofacial structures in B-RPE, whereas it was loaded by the teeth indirectly in C-RPE. Higher stress in B-RPE also presented a far-reaching feature by transmit stresses via frontomaxillary and zygomaticofrontal sutures to frontal bone, and via the zygomaticotemporal sutures to temporal bone (Fig. 6a–f). Moreover, the concentrated stresses in the pterygoid areas were posteriorly spread from sphenoid body to the lateral margin of foramen magnum in B-RPE, but the physical feature was minimal in C-RPE (Fig. 6g–h). In C-RPE, among all the craniofacial sutures, zygomaticofrontal sutures experienced the highest stress, followed by pteryomaxillary sutures, and the nasomaxillary suture was the minimal. However, in B-RPE, pteryomaxillary sutures experienced the highest stress and the minimal stress was concentrated on the zygomaticomaxillary sutures. Whether in C-RPE or B-RPE, the magnitudes of concentrated stresses on different bony structures and sutures were greater on cleft side than those on non-cleft side, especially in the posterior area of skull (Table 6; Fig. 7a–b).

**Table 4:** Stress distributions (MPa) at the palatal and tooth landmarks after activation of the device in the different models

Positions	C-RPE		B-RPE	
	Cleft side	Non-cleft side	Cleft side	Non-cleft side
*Palatal landmarks*				
Anterior palate (A1)	0.2562	0.0047	0.0088	0.0164
Middle palate (A2)	0.2176	0.0152	1.1956	0.0175
Posterior palate (A3)	0.0014	0.0012	0.0272	0.0125
*Dental landmarks*				
Cusp tip of 4 (B1)	0.3461	0.2765	0.0009	0.0005
Cusp tip of 5 (B2)	0.0035	0.0012	0.0010	0.0006
Cusp tip of 6 (B3)	0.1978	0.5720	0.0042	0.0064
Cusp tip of 7 (B4)	0.0034	0.0059	0.0033	0.0089

**Table 5:** Stress distributions (MPa) at the palatal and tooth landmarks after activation of the device in the different models

Positions	Type A		Type B	
	Cleft side	Non-cleft side	Cleft side	Non-cleft side
*Medial pterygoid*				
Superior (C)	0.1051	0.1411	0.1446	0.4484
Inferior(D)	2.7455	5.4822	9.4765	15.1840
*Lateral pterygoid*				
Superior (E)	0.2582	0.2964	0.8974	1.5393
Inferior(F)	2.6852	4.3364	9.3405	14.2250
*Maxilla*				
Maxillary tuberosity(G)	0.1028	0.1692	0.2693	0.3775
Anterolateral wall(H)	0.9878	1.0757	2.1953	1.9032
Inferior orbital rim(I)	0.1794	0.1265	0.3481	0.2525
Frontal process(J)	3.4256	2.7213	8.6443	5.6707
Anterior nasal spine(K)	0.4898	0.0055	0.0131	0.0104
*Nasal cavity*				
Superior rim (L)	1.4574	0.7521	7.4482	2.7186
Inferior rim(M)	0.3133	0.3257	1.4511	0.5243
*Zygomatic bone*				
Frontal process (N)	0.9418	1.0504	1.7545	3.2375
Temporal process(O)	0.4555	0.6365	0.5327	1.7492
Maxillary process(P)	0.3717	0.8453	1.0741	0.6540

**Table 6:** Stress distributions (MPa) at the craniofacial sutures after activation of the device in the different models

Positions	Type A		Type B	
	Cleft side	Non-cleft side	Cleft side	Non-cleft side
Nasomaxillary sutures	0.1091	0.0992	0.4039	0.4107
Frontomaxillary sutures	0.2898	0.2941	0.7361	0.6765
Zygomaticomaxillary sutures	0.1764	0.1507	0.4026	0.3648
Zygomaticofrontal sutures	0.9044	0.6037	2.0310	1.5310
Zygomaticotemporal sutures	0.6008	0.3222	1.4347	0.8118
Pteryomaxillary sutures	0.6604	0.6280	2.9119	2.1126

## Disscusion

Patients with UCLP usually undergo surgery repair in the early ages, but the postsurgical complications caused by the scar tissue contractions surrounding the musculature are various and common[2]. The most challenging issue in patients with UCLP is the maxillary dysplasia, which grows in a retracted and asymmetrical pattern. The conventional HYRAX expander (C-RPE) is an effective treatment for the correction of transverse discrepancies, which is routinely applied combining with the protraction of retracted maxilla in adolescence with UCLP[21]. However, Lucas Cardinal demonstrated that the C-RPE could induce an average of 0.8 mm decrease in buccal bone thickness, and a 0.5 mm increase in dehiscence[7]. Those tooth effect of C-RPE wound be enhanced in late adolescence[3]. By contrast, the bone-borne palatal expander (B-RPE) has been proved to be effective for arch expansion in adolescent or adult patients without CLP, which produces more skeletal expansion and less buccal inclination of teeth[22, 23]. Moreover, unlike the C-RPE, the alignment of dentition with brackets could be carried out synchronously using B-RPE[2]. Nevertheless, a precise evaluation about the biomechanical effects of bone anchoring expansion on late adolescence with UCLP is rare at present. It is possible that the biomechanical mechanism and effects of bone anchoring expansion are particularly different due to the lack of integrity of palate and the deformation of surrounding anatomic structures[20]. This study is novel because it is the first to evaluate and compare the biomechanical effects of C-RPE and B-RPE on the whole craniofacial structures in the late adolescence with UCLP.

Previous studies reported that the magnitude of force generated by expansion device should be highly up to 120 N to activate the separation of palatal suture in normal population[24]. Since the palatal suture is missing and the transversal stability of nasomaxillary complex is reduced in patients with UCLP, the produced force by expansion appliance is necessary to be attenuated. Thus, Haofu Lee used 700-1100 g (Equivalent force: 6.86-10.78 N) in their finite element study to simulate the 5 mm expansion of dental arch in a 7-year-old girl with UCLP[20]. By conducting a comparative biomechanical analysis of maxillary, Christof Holberg demonstrated that an expansion force of 2 N was sufficient to induce the skeletal effect on maxilla in patient with UCLP[24]. However, the differences in appliances, patient ages, maturity of craniofacial sutures, shape and density of palate, might cause various biomechanical results when applying different expansion force[10]. Hence, different from previous studies, the expanders in present study were activated transversely by 0.5 mm to simulate the clinical daily expansion as in the non-cleft model[10].

In this study, the expander was anchored by mini-screws in palatal slope, which was different from the non-cleft patient with the miniscrews symmetrically inserted 3 mm to the midpalatal suture [11, 13]. In non-cleft patients, the locations of miniscrews in B-RPE were mostly distributed from the middle to posterior palate, regions with decreased alveolar height and closing to the midpalatal suture [13, 25]. However, in UCLP patients, the horizontal area of palatal bone was deformed in morphology and limited in the thickness around posterior region, causing the result that the implantation sites of minicrews were inconsistent among patients[26]. To ensure fixation, the four implants in this study were inserted into the palatal slope, 8 mm beneath the alveolar ridge as reported by Park, J. H. [19]. Jin-Young Choi demonstrated that the B-RPE with anchor screws placed at different heights below the cementoenamel junction, wound generate significant changes in displacement and stress distribution around paramedian area[8]. Because of the asymmetrical palatal slopes in three-dimension, four miniscrews were implanted in different vertical and sagittal distances, and this individual expansion device might reduce the transversal effect, but enhance the vertical movement. Actually, in this UCLP model, although the posterior palate on cleft side was downwardly deformed, the B-RPE could significantly enhance the transverse to vertical effect ratio when compared with the C-RPE (Cleft side: C-RPE = 0.0698/0.0515 VS B-RPE = 0.2085/0.1206. Non-cleft side: C-RPE = 0.0595/0.0401 VS B-RPE = 0.1960/0.0906). This individual design in B-RPE contributed to correct the vertical deformation of the palatal fornix.

Compared with the C-RPE, B-RPE generated increased skeletal expansion, which agreed with the previous studies in non-cleft patients[9, 27]. The anchoring rings on the first molar experienced highest stress concentration, which was nearly twice as greater as the maximum stress concentrated on the implantation site. Moreover, in C-RPE, the maximum expanding stress on the supporting molar was obviously greater on non-cleft side than it on cleft side, while it was relatively equal between the bilateral sides in B-RPE. By contrast, in non-cleft patient, Hartono, N found that B-RPE resulted in higher stress values (328.65 MPa) around the implantation sites than the C-PRE on the anchoring teeth (17.732 MPa) [16]. Lee, S. C demonstrated that the B-RPE, an operation assisted with surgical separation along the midsagittal direction, wound enhance the stresses in various structures, including the mini-implants, the maxillofacial sutures and the zygomatic arch [10]. Those alterations might be caused by the diminished resistance to expansion for the absence of palatal suture in non-cleft patient [10]. For the UCLP patient, the palate was congenitally separated, and the expanding force could be transmitted more efficiently to the surrounding craniofacial structures. Thus, the stresses experienced on the pterygomaxillary, zygomaxillary, and nasomaxillary buttresses were generally greater than those in non-cleft patient [8, 27]. In this study, using the late adolescence with UCLP, we directly loaded the expanding force on the basal bone for the first time. Our results indicated that the buccal alveolar in B-RPE experienced less compressive force than it in C-RPE, which was assistant to reduce the risk of bone fenestration and dehiscence [7, 21]. Previous studies showed that the pterygomaxillary sutures offered primary anatomic resistance to expansion in non-cleft patients, which could be reduced by the LeFort I and paramedian osteotomies, as well as the pterygomaxillary separation [5, 28]. In UCLP patients applied with B-RPE, the expanding force from anchoring miniscrews was dispersed posteriorly to disarticulate the maxilla from the neighboring pterygoid of sphenoid. Thus, miniscrew assisted non-surgical palatal expansion was effective in adults [22]. Sutures play a functional role in osteogenesis, including connection and cushion. Oliveira CB demonstrated that the midpalatal and circummaxillary sutures wound become more rigid as aging progresses, which might be the reason of unsuccessful expansion in late adolescence [29]. As for UCLP patients, Wu previously established the three-dimensional model of cranio-maxillary complex with sutures, and validated the effectiveness using FEA [30]. In this study, the zygomaticofrontal, zygomaticotemporal and pteryomaxillary sutures experienced more stress than other sutures when expanding with B-RPE. Seong Cheon Lee [10] found that the expansion force from C-RPE was mostly concentrated on the zygomaticomaxillary sutures, which was significantly reduced when using B-RPE. On the contrary, the stresses in frontomaxillary and frontozygomatic sutures were obviously increased using B-RPE, which cloud be furtherly enhanced when assisted with mid-palatal suture osteotomy. Thus, stress distributing patterns on circum-maxillary sutures were obviously different between the C-RPE and B-RPE, and the absent of midpalatal suture might be the important contribution to this discrepancy.

In this study, compared with the C-RPE, the palatoalvolar and other craniofacial structures also indicated considerable increase in dispalcement using the B-RPE, especially around the regions of inferior maxilla, dental arch and pterygoid plates of sphenoid bone. The maximum total displacement generated by B-RPE occurred in the first pre-molar on cleft side, greater than it in C-RPE. Nata´lia Costa Veloso conducted a retrospective investigation basing on the analysis of CBCT images from 40 adolescences with UCLP, and the result indicated that maxillary changes in C-RPE were restricted to the dentoalveolar region [18]. This result was consistent with our finding. Actually, as reported by H.W. Moon in non-cleft patients [13], the expansion of nasomaxillary complex proceeded in V-shaped patterns in the horizontal and vertical directions. In this study, whether on cleft or non-cleft side, we also found that the amount of anterior expansion of palate was larger than the posterior, and the mount of inferior rim of nasal cavity was larger than the superior rim. In addition, the displacement of frontonasal suture was minimal, which highly supported the clinical conclusion of Moon, who suggested that the center of resistance of the maxilla was at the midpoint of frontonasal suture [13]. In addition, unlike the non-cleft patients in sagittal direction, the minor segment of the maxilla in UCLP patients treated with RPE was commonly associated with mediolingual rotation. Thus, the vector of the distraction was suggested to be oblique to the midpalatal plane, which was beneficial for obtaining more advancement in minor segment [20, 31]. Interestingly, in this study, although the expander in B-RPE was not in an oblique direction, the anterior displacement in dental arch and palate on cleft side was significantly increased, while the amount on non-cleft side was largely decreased compared with the C-RPE, indicating the superiority in the correction of unilateral maxillary dysplasia and crossbite.

The biomechanical mechanism and effects of FEA are highly dependent on the quality of the constructed models to simulate the real structure, which can be influenced by the number of elements. In this study, a three dimensional finite element model was consisted of 1,186,791 tetrahedral elements and 1,806,003 nodes, which was far exceeding other relating studies [10, 18, 20]. However, there were still several limitations in this study. First, only one patient with UCLP was selected for model establishing, which was definitely insufficient to simulate the variety of clinical deformations in craniofacial structures. Second, when modeling, the trabecular and soft tissue were not taken into consideration as previous studies [18, 20]. Actually, the simplified material properties of craniofacial tissues were not homogeneous and elastic, which wound influence the stimulating result of deformation and equivalent von-mises stress. Third, the extent of the cleft and availability of palatal bone wound definitely influence the placement sites for bands or mini-screws. Different positons of anchoring teeth on alveolar ridge or mini-screws on palatal slope could result in different outcomes in the transmission and dissipation of expanding force. For feasibility considerations, we designed the appliances according to the clinical situation of single patient. Thus, the present results only made initial conclusions about the discrepancies between the C-RPE and B-RPE. Last, since the expanders in our study were activated at 0.25 mm only (0.125 mm per side), the deformity in the suture area might be limited. Further investigations of force applications and creep strains over longer time periods will be helpful to mimic the clinical scenario. In the future, a comparison using the clinical cases is necessary to furtherly identify the outcomes of this study.

## Conclusions

1. In late adolescence with UCLP, compared with the conventional tooth-borne expander, bone-borne maxillary expander transmits more transverse expansion forces to surrounding craniofacial structures, while the stresses in periodontal ligament and buccal alveolar are significantly decreased, which is beneficial to avoid the unexpectable alveolar fenestration.
2. Compared with the conventional tooth-borne expander, bone-borne maxillary expander contains more advantages in correcting the mediolingual retraction of minor segment of maxilla, without over displacement in the major segment. Bone-borne maxillary expander is also helpful to correct the vertical deformation of the palatal fornix in posterior region.
3. Bone-borne maxillary expander can enhance the skeletal expansion at the alveolar level and diminish the dental effect, which may serve as an effective device for the nonsurgical treatment of transverse maxillary deficiency in late adolescence with UCLP.

## Data Availability

All data generated or analysed during this study are included in this published article.

## References

[1] Haque S, Khamis MF, Alam MK, Ahmad W (2020). Effects of multiple factors on treatment outcome in the Three-Dimensional Maxillary Arch Morphometry of Children with Unilateral Cleft lip and palate. J Craniofac Surg.

[2] Scolozzi P, Verdeja R, Herzog G, Jaques B (2007). Maxillary expansion using transpalatal distraction in patients with unilateral cleft lip and palate. Plast Reconstr Surg.

[3] Lin L, Ahn HW, Kim SJ, Moon SC, Kim SH, Nelson G (2015). Tooth-borne vs bone-borne rapid maxillary expanders in late adolescence. Angle Orthod.

[4] Veloso NC, Mordente CM, de Sousa AA, Palomo JM, Yatabe M, Oliveira DD, Souki BQ, Andrade I (2020). Three-dimensional nasal septum and maxillary changes following rapid maxillary expansion in patients with cleft lip and palate. Angle Orthod.

[5] Gautam P, Zhao L, Patel P (2011). Determining the osteotomy pattern in surgically assisted rapid maxillary expansion in a unilateral palatal cleft: a finite element model approach. Angle Orthod.

[6] Carlson C, Sung J, McComb RW, Machado AW, Moon W, Microimplant-assisted rapid palatal expansion appliance to orthopedically correct transverse maxillary deficiency in an adult, American journal of orthodontics and dentofacial orthopedics: official publication of the American Association of Orthodontists, its constituent societies, and the American Board of Orthodontics 149(5) (2016) 716 – 28. 10.1016/j.ajodo.2015.04.043 27131254

[7] Cardinal L, Rosa Zimermann G, Mendes FM, Andrade I Jr, Oliveira DD, Dominguez GC. Dehiscence and buccal bone thickness after rapid maxillary expansion in young patients with unilateral cleft lip and palate, american journal of orthodontics and dentofacial orthopedics: official publication of the American Association of Orthodontists, its constituent societies. and the American Board of Orthodontics; 2022. 10.1016/j.ajodo.2021.01.038 35153114

[8] Choi JY, Choo H, Oh SH, Park JH, Chung KR, Kim SH, Finite element analysis of C-expanders with different vertical vectors of anchor screws, American journal of orthodontics and dentofacial orthopedics: official publication of the American Association of Orthodontists, its constituent societies, and the American Board of Orthodontics 159(6) (2021) 799–807. 10.1016/j.ajodo.2020.02.024 33762139

[9] Lagravère MO, Carey J, Heo G, Toogood RW, Major PW, Transverse, vertical, and anteroposterior changes from bone-anchored maxillary expansion vs traditional rapid maxillary expansion: a randomized clinical trial, American journal of orthodontics and dentofacial orthopedics: official publication of the American Association of Orthodontists, its constituent societies, and the American Board of Orthodontics 137(3) (2010) 304.e1-12; discussion 304-5. 10.1016/j.ajodo.2009.09.016 20197161

[10] Lee SC, Park JH, Bayome M, Kim KB, Araujo EA, Kook YA, Effect of bone-borne rapid maxillary expanders with and without surgical assistance on the craniofacial structures using finite element analysis, American journal of orthodontics and dentofacial orthopedics: official publication of the American Association of Orthodontists, its constituent societies, and the American Board of Orthodontics 145(5) (2014) 638 – 48. 10.1016/j.ajodo.2013.12.029 24785928

[11] Seong EH, Choi SH, Kim HJ, Yu HS, Park YC, Lee KJ (2018). Evaluation of the effects of miniscrew incorporation in palatal expanders for young adults using finite element analysis. Korean J Orthod.

[12] Pan X, Qian Y, Yu J, Wang D, Tang Y, Shen G (2007). Biomechanical effects of rapid palatal expansion on the craniofacial skeleton with cleft palate: a three-dimensional finite element analysis, the cleft palate-craniofacial journal: official publication of the american Cleft. Palate-Craniofacial Association.

[13] Moon HW, Kim MJ, Ahn HW, Kim SJ, Kim SH, Chung KR, Nelson G (2020). Molar inclination and surrounding alveolar bone change relative to the design of bone-borne maxillary expanders: a CBCT study. Angle Orthod.

[14] Papathanasiou E, Trotman CA, Scott AR, Van Dyke TE (2017). Current and emerging treatments for Postsurgical Cleft Lip Scarring: effectiveness and mechanisms. J Dent Res.

[15] Provatidis C, Georgiopoulos B, Kotinas A, McDonald JP (2007). On the FEM modeling of craniofacial changes during rapid maxillary expansion. Med Eng Phys.

[16] Hartono N, Soegiharto BM, Widayati R (2018). The difference of stress distribution of maxillary expansion using rapid maxillary expander (RME) and maxillary skeletal expander (MSE)-a finite element analysis. Prog Orthodont.

[17] Lee HK, Bayome M, Ahn CS, Kim SH, Kim KB, Mo SS, Kook YA (2014). Stress distribution and displacement by different bone-borne palatal expanders with micro-implants: a three-dimensional finite-element analysis. Eur J Orthod.

[18] Mathew A, Nagachandran KS, Vijayalakshmi D (2016). Stress and displacement pattern evaluation using two different palatal expanders in unilateral cleft lip and palate: a three-dimensional finite element analysis. Prog Orthodont.

[19] Park JH, Bayome M, Zahrowski JJ, Kook YA, Displacement and stress distribution by different bone-borne palatal expanders with facemask: A 3-dimensional finite element analysis, American journal of orthodontics and dentofacial orthopedics: official publication of the American Association of Orthodontists, its constituent societies, and the American Board of Orthodontics 151(1) (2017) 105–117. 10.1016/j.ajodo.2016.06.026 28024761

[20] Lee H, Nguyen A, Hong C, Hoang P, Pham J, Ting K, Biomechanical effects of maxillary expansion on a patient with cleft palate: A finite element analysis, American journal of orthodontics and dentofacial orthopedics: official publication of the American Association of Orthodontists, its constituent societies, and the American Board of Orthodontics 150(2) (2016) 313 – 23. 10.1016/j.ajodo.2015.12.029 PMC5557378 27476365

[21] Singh S, Batra P, Raghavan S, Sharma K, Srivastava A (2022). Evaluation of Alt-RAMEC with Facemask in patients with unilateral cleft lip and palate (UCLP) using cone Beam Computed Tomography (CBCT) and finite element Modeling-A clinical prospective study, the cleft palate-craniofacial journal: official publication of the american Cleft. Palate-Craniofacial Association.

[22] Winsauer H, Walter A, Katsaros C, Ploder O (2021). Success and complication rate of miniscrew assisted non-surgical palatal expansion in adults - a consecutive study using a novel force-controlled polycyclic activation protocol. Head Face Med.

[23] Tehranchi A, Ameli N, Najirad Z, Mirhashemi FS (2013). Comparison of the skeletal and dental changes of tooth-borne vs. bone-borne expansion devices in surgically assisted rapid palatal expansion: a finite element study. Dent Res J.

[24] Holberg C, Holberg N, Schwenzer K, Wichelhaus A, Rudzki-Janson I (2007). Biomechanical analysis of maxillary expansion in CLP patients. Angle Orthod.

[25] Cantarella D, Dominguez-Mompell R, Moschik C, Sfogliano L, Elkenawy I, Pan HC, Mallya SM, Moon W (2018). Zygomaticomaxillary modifications in the horizontal plane induced by micro-implant-supported skeletal expander, analyzed with CBCT images. Prog Orthodont.

[26] Rusková H, Bejdová S, Peterka M, Krajíček V, Velemínská J (2014). 3-D shape analysis of palatal surface in patients with unilateral complete cleft lip and palate, Journal of cranio-maxillo-facial surgery: official publication of the European Association for Cranio-Maxillo. -Facial Surg.

[27] Yoon S, Lee DY, Jung SK (2019). Influence of changing various parameters in miniscrew-assisted rapid palatal expansion: a three-dimensional finite element analysis. Korean J Orthod.

[28] Han UA, Kim Y, Park JU (2009). Three-dimensional finite element analysis of stress distribution and displacement of the maxilla following surgically assisted rapid maxillary expansion, Journal of cranio-maxillo-facial surgery: official publication of the European Association for Cranio-Maxillo. -Facial Surg.

[29] Oliveira CB, Ayub P, Angelieri F, Murata WH, Suzuki SS, Ravelli DB, Santos-Pinto A (2021). Evaluation of factors related to the success of miniscrew-assisted rapid palatal expansion. Angle Orthod.

[30] Wu ZF, Lei YH, Li WJ, Liao SH, Zhao ZJ (2013). Construction and validation of a three-dimensional finite element model of cranio-maxillary complex with sutures in unilateral cleft lip and palate patient. Shanghai J stomatology.

[31] Kuroe K, Iino S, Shomura K, Okubo A, Sugihara K, Ito G (2003). Unilateral advancement of the maxillary minor segment by distraction osteogenesis in patients with repaired unilateral cleft lip and palate: report of two cases, the cleft palate-craniofacial journal: official publication of the american Cleft. Palate-Craniofacial Association.

